# SUMOylation regulates the nuclear mobility of CREB binding protein and its association with nuclear bodies in live cells

**DOI:** 10.1016/j.bbrc.2009.12.040

**Published:** 2010-01-01

**Authors:** Colm M. Ryan, Karin B. Kindle, Hilary M. Collins, David M. Heery

**Affiliations:** Gene Regulation Group, Centre for Biomolecular Sciences, School of Pharmacy, University of Nottingham, University Park, Nottingham NG7 2RD, United Kingdom

**Keywords:** SUMO, small ubiquitin like modifier, CBP, CREB binding protein, YFP/GFP, yellow/green fluorescent protein, PML, promyelocytic leukaemia, AML, acute myeloid leukaemia, TDG, thymine DNA glycosylase, HDAC, histone deacetylase, FRAP, fluorescence recovery after photo-bleaching, CRD, cell cycle repression domain, CBP, SUMOylation, FRAP, PML, AML1, Nuclear bodies

## Abstract

The lysine acetyltransferase CREB binding protein (CBP) is required for chromatin modification and transcription at many gene promoters. In fixed cells, a large proportion of CBP colocalises to PML or nuclear bodies. Using live cell imaging, we show here that YFP-tagged CBP expressed in HEK293 cells undergoes gradual accumulation in nuclear bodies, some of which are mobile and migrate towards the nuclear envelope. Deletion of a short lysine-rich domain that contains the major SUMO acceptor sites of CBP abrogated its ability to be SUMO modified, and prevented its association with endogenous SUMO-1/PML speckles *in vivo*. This SUMO-defective CBP showed enhanced ability to co-activate AML1-mediated transcription. Deletion mapping revealed that the SUMO-modified region was not sufficient for targeting CBP to PML bodies, as C-terminally truncated mutants containing this domain showed a strong reduction in accumulation at PML bodies. Fluorescence recovery after photo-bleaching (FRAP) experiments revealed that YFP–CBPΔ998–1087 had a retarded recovery time in the nucleus, as compared to YFP–CBP. These results indicate that SUMOylation regulates CBP function by influencing its shuttling between nuclear bodies and chromatin microenvironments.

## Introduction

CBP is a large multi-domain nuclear protein that interacts with and acetylates histones and other nuclear proteins [1]. This facilitates its function as a coactivator of gene transcription, as recruitment of CBP to active promoters correlates with histone hyperacetylation and gene activation. Recruitment of CBP to active gene promoters/enhancers is transient and often dependent on an activating signal, e.g. at genes that are induced by signal transduction events or hormone ligands. This suggests that CBP mobility in the nucleus is highly dynamic, and thus tightly regulated. Antibody staining of CBP in fixed cells reveals that a large proportion of nuclear CBP is localised in nuclear bodies (also called PML bodies) [2] from where it shuttles to sites of transcription. Currently, little is known regarding how this is achieved, although it is likely to be dependent on protein–protein interactions, and post-translational modification of CBP.

CBP is subjected to a range of post-translational modifications including acetylation [3], phosphorylation [4–7], ubiquitylation [8,9] and SUMOylation [10] which regulate its functional interactions, activity and stability. SUMOylation of CBP occurs within a region containing eight potential SUMO acceptor sites (amino acids 998–1090), and mediates interaction of CBP with the transcriptional corepressors Daxx and HDAC6 [10–12]. Our studies have shown that interactions between CBP and the leukaemogenic fusion protein MOZ-TIF2 [13] or cytoplasmic isoforms of PML [14] can sequester it away from nuclear bodies, and that in the case of MOZ-TIF2, CBP binding is necessary for leukaemogenesis [13]. Thus, dynamic association with nuclear bodies appears to be an important aspect of the normal function of CBP. In this study we investigate the localisation of YFP–CBP to nuclear bodies in live cells, and investigate the role of the SUMOylated domain of CBP in this process.

## Materials and methods

*Expression and reporter plasmids.* The following expression and reporter plasmids were gifts; pCMV5 AML1b (D.E. Zhang), pT109-luc (A. Zelent), pSG5 HA-SUMO-1 (V. Di Laurenzo), HA-TDG (S. Ali), pEGFP SUMO-1, pEGFP-SUMO-1(G/A) (J. Palvimo) and pcDNA3.1-FLAG-CBP (E. Kalkhoven). The β-galactosidase reporter pCH110 (Promega) was used as an internal control in reporter assays. YFP–CBP was generated by subcloning a B*am*HI fragment containing full-length mouse CBP cDNA from pRSV-CBP-HA (R. Goodman) into pEYFP-C1 (*Clontech*). Recombinant PCR was used to introduce a deletion of the sequence encoding amino acids 998–1087. The vector pcDNA3.1-FLAG-CBP was used to generate the deletion series pcDNA3.1-FLAG-CBP 1–507, 1–1100, 1–1458 and 1–1901 by digestion with *Bln*I, *Xba*I, *Xho*I and *Xma*I restriction endonucleases, respectively, followed by gel purification and re-ligation of the appropriate fragments. All deletion boundaries or PCR generated constructs were verified by sequencing.

*Reporter assays*. COS-1 cells were maintained in Dulbecco’s modified eagle medium (DMEM) supplemented with 10% foetal calf serum (FCS) and 2 mM glutamine at 5% CO_2_. AML1 reporter assays, were performed essentially as described previously [23] using 100 ng β-galactosidase reporter pCH110, 500 ng of the AML1-responsive reporter pT109-luc, 100 ng AML1 and 500 ng of the other expression vectors along with empty vector to standardise the quantity of transfected DNA in each well. The luciferase activity data was normalised for β-galactosidase activity.

*In vitro SUMOylation assay.* The expression constructs used in the SUMOylation assay were *in vitro* transcribed and translated using TNT coupled reticulolysate system (Promega) to produce ^35^S-methionine labelled proteins. Reaction mixtures contained equal amounts of the ^35^S-labelled IVT protein, recombinant SAE1/SAE2 (0.1 μg), Ubc9 (0.45 μg), SUMO-1 or SUMO-2 (1 μg) (Alexis Biochemicals) in SUMO conjugation buffer (50 mM Tris pH 7.5, 10 mM MgCl_2_, 5 mM ATP, 0.2 mM DTT), and were incubated at 30 °C for 2 h. The reactions were stopped by the addition of 4XSDS-loading buffer and incubated at 95 °C for 5 min. The proteins were resolved on 6% or 10% SDS–PAGE gels and proteins visualised using a phosphorimager.

*Immunofluorescence*. Transfections were performed using TransFast (Promega). Immunostaining of fixed cells was performed as previously described [13]. Fixed cells were stained with primary antibodies for 1 h (1:50 dilution for overexpressed proteins, 1:10 for CBP, 1:30 for PML, 1:100 for SUMO-1 and 1:30 for FLAG). After washing to remove unbound primary antibody, cells were incubated with the secondary antibody (1:400 dilution). The samples were viewed with a fluorescence microscope (Zeiss Axiovert 200 M) or a confocal laser scanning microscope (Zeiss LSM510).

*Live cell imaging.* Cells were seeded at a density of 5 × 10^5^ cells on 22-mm-diameter circular coverslips for 24 h, followed by transfection with 1 μg of pEYFP–CBP. At 14 h post-transfection, the coverslip was mounted in a steel coverslip holder in CO_2_-independent medium (Invitrogen) supplemented with l-glutamine and overlaid with mineral oil (Sigma). The coverslip holder was inserted in a Patch Slice MicroIncubator, which was kept at a constant temperature of 37 °C by a TC-202A temperature controller (Digitimer). After the microincubator was mounted on the microscope stage, images were taken at 5-min intervals with an ORCA ER charge-coupled-device camera (Hamamatsu) attached to a Nikon TE300 inverted microscope. Five optical sections (*z*-sections) with 0.1 μm intervals, obtained with a high-speed Piezo focus drive (Orbit II) fixed to a 60×, 1.4-numerical-aperture objective, were captured at the individual time points by using Openlab 3.09 software (Improvision). The individual sections were merged by using the Openlab software, processed with Adobe Photoshop, and saved as a quick-time movie. Stills were made with Volocity software (Improvision).

*Fluorescence recovery after photo-bleaching (FRAP).* Cells were seeded and observed in 8 well LabTek chamber slides (Nunc) in DMEM, supplemented with 10% foetal calf serum and 1% l-Glutamine. FRAP experiments were performed 24 h post-transfection (250 ng of YFP–CBP or YFP–CBPΔ998–1087 expression plasmids). Bleaching was performed using a 488 nm laser at 100% output for 1.3 s and 100 iterations were performed to optimise bleaching of the selected region of interest (ROI). Nucleolar regions were excluded in the choice of ROIs. The laser output was attenuated to 1% output for imaging to minimise bleaching and phototoxicity. Single *z*-sections were collected in 12-bit format before bleaching, directly after and every 10 s thereafter for 2 min. Mean fluorescent intensities of chosen ROIs were determined using the LSM software and data was exported to Excel (Microsoft Inc.) for analysis. Curves were fitted using Sigma Plot 11.0 and Standard Error of the Mean is shown for the indicated time points.

## Results

### CBP nuclear bodies are mobile and can migrate toward the nuclear periphery

**Fig. 1:**
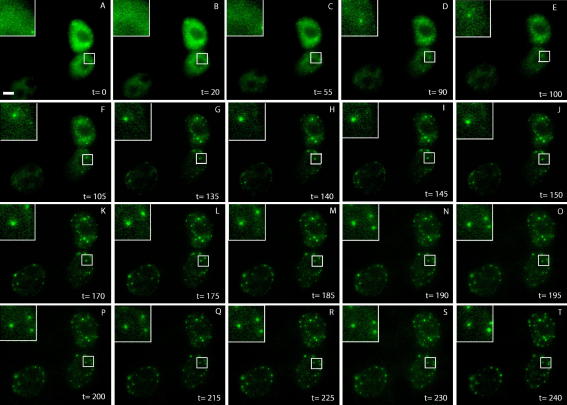
*Time-lapse imaging of YFP–CBP*. HEK293 cells were transfected with 1 μg pEYFP–CBP expression plasmid. Image capture was initiated approximately 14 h post-transfection at 5 min intervals for 4 h. The images shown were deconvolved and merged to produce the composite images. The insets in the top left hand corner show a higher magnification of the selected region, highlighting the formation of CBP-containing nuclear bodies and their relative migration during the experiment.

To study the subcellular localisation of CBP in live cells, HEK293 cells were transiently transfected with YFP–CBP expression plasmids [13] and monitored 12–18 h post-transfection. Cells showing moderate levels of YFP–CBP expression were chosen for time-lapse imaging. As shown in Fig. 1, at approximately 14 h post-transfection (designated *t* = 0) YFP–CBP proteins displayed a diffuse nuclear distribution, excluded from nucleoli. However, time-lapse imaging for a further 4 h revealed gradual accumulation of YFP–CBP in nuclear bodies. This gradual accumulation of nascent YFP–CBP proteins in nuclear bodies suggests it may be dependent on protein–protein interactions, post-translational modifications or turnover of CBP proteins not targeted to nuclear bodies. Viewed as a time-lapse movie, it was observed that some YFP–CBP speckles showed considerable mobility within the nucleus, whereas other speckles appear to remain largely static. This is highlighted in the insets (Fig. 1G–T, top left corners) where some speckles (e.g. Fig. 1G) remain relatively unchanged during image capture, whereas others appear to move distances of at least 1 μm (e.g. compare with Fig. 1L–T). Similar observations have been reported previously for another PML body component, i.e. SP100, where a proportion of GFP-SP100-containing nuclear speckles were found to move in an ATP-dependent manner [15].

### SUMOylation-defective CBP does not accumulate in PML bodies

**Fig. 2:**
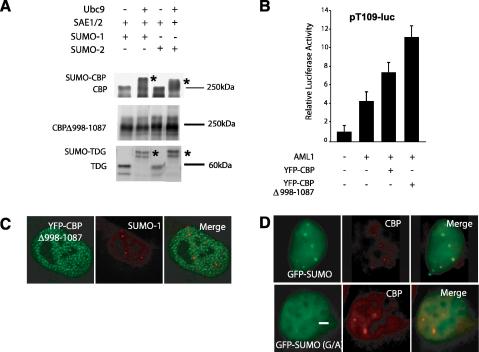
(A) SUMO-defective CBP. Mouse CBP, CBPΔ998–1087 and TDG were *in vitro* translated in the presence of [^35^S]-methionine to give full-length radio-labelled proteins. The IVT proteins were then used for *in vitro* SUMO assays using recombinant Ubc9, SAE1/SAE2 and SUMO-1 or SUMO-2, as indicated. The proteins were resolved via SDS–PAGE prior to autoradiography. SUMOylated CBP and TDG proteins are indicated by asterices. (B) Deletion of the residues 998–1087, corresponding to the cell cycle regulatory domain (CRD) of p300 (11), enhances CBP coactivation of AML1. Transient transfection experiments using the AML1-responsive luciferase reporter pT109-luciferase. The effect of the indicated CBP or CBPΔ998–1087 constructs on AML1-mediated reporter activation is shown. The data is represented as fold induction luciferase reporter activity (normalised to β-galactosidase) over the control (no AML1). A representative experiment is shown, with standard errors of the mean from triplicate samples. (C) Mislocalisation of a SUMO-defective CBP. Distribution of YFP–CBPΔ998–1087 in fixed COS-1 cells (left panel,) immunostained for endogenous SUMO-1 proteins (middle panel). (D) Detection of GFP-SUMO-1 or non-conjugable GFP-SUMO-1 G/A mutant proteins in COS-1 cells. After fixing, cells were immunostained for endogenous CBP to label nuclear bodies (middle panels). Merged images are also shown (right panels).

CBP is SUMOylated within a region containing a cluster of lysine residues that is partially conserved within p300 [10,11]. We generated a construct in which this region was removed (YFP–CBPΔ998–1087). *In vitro* assays confirmed that while full-length CBP or thymine DNA glycosylase (TDG) proteins can be efficiently conjugated to SUMO-1 or SUMO-2 in a Ubc9-dependent manner, the YFP–CBPΔ998–1087 mutant did not show any mobility shift due to SUMOylation in these assays (Fig. 2A). This confirms that the YFP–CBPΔ998–1087 mutant is defective for SUMOylation.

However, deletion of the SUMOylated region did not affect the ability of CBP to localise to the nucleus (Figs. 2C and 4A) or its function as a coactivator. Indeed, reporter assays revealed that YFP–CBPΔ998–1087 displayed an enhanced ability to stimulate AML1-mediated reporter activation. As shown in Fig. 2B, expression of AML1 alone led to an approximately fourfold increase in reporter activation, which increased to eightfold upon co-expression with YFP–CBP. However, the reporter was activated 12-fold by co-expression of AML1 and YFP–CBPΔ998–1087 (Fig. 2B), despite being expressed to similar levels as determined by western blots (data not shown). This result is consistent with other reports that SUMOylation of p300 and CBP may suppress coactivator activities, possibly through recruitment of HDAC complexes [12], and that a SUMO-defective CBP showed increased coactivator activity in IRF1 reporter assays [10].

To determine whether YFP–CBPΔ998–1087 can localise to PML bodies, we expressed it in COS-1 cells. As shown in Fig. 2C, YFP–CBPΔ998–1087 was detected in large numbers of smaller foci (microspeckles) in approximately 90% of transfected (fixed) cells. Co-staining of these cells with α-SUMO-1 to reveal nuclear bodies showed that the YFP–CBPΔ998–1087 microspeckles are distinct from nuclear bodies (Fig. 2C). To confirm that SUMO-1 accumulates in PML bodies, we expressed GFP-SUMO or a GFP-SUMO (G/A) mutant that cannot undergo conjugation to acceptor proteins [16]. The GFP-SUMO was found to accumulate in PML bodies, as revealed by staining for endogenous nuclear CBP (Fig. 2D). Note that endogenous PML and CBP proteins show perfect colocalisation in these experiments ([18] and data not shown). However, unlike GFP-SUMO, GFP-SUMO (G/A) revealed a diffuse nuclear pattern that was not enriched in PML bodies (Fig. 2D). Quantitation of subcellular distribution phenotypes in transfected cell nuclei (*n* = 100) revealed that GFP-SUMO was detected in nuclear speckles in 90% or 68% of COS-1 or HEK293 cells, respectively, compared with 10% or 0%, respectively for GFP-SUMO(G/A) (data not shown).

### The C-terminus of CBP is necessary for efficient SUMOylation and targeting to nuclear bodies

**Fig. 3:**
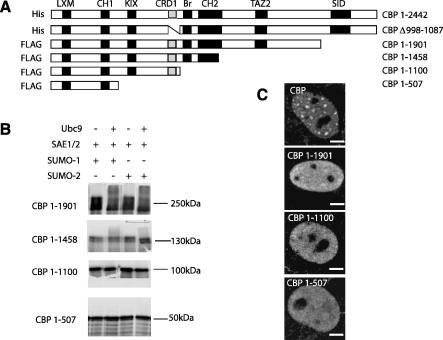
*Sequences outside the CRD are required for efficient SUMOylation of CBP and targeting to nuclear bodies*. (A) Schematic representation showing the domain structure of full-length CBP, CBPΔ998–1087 and CBP C-terminal deletion mutants used. (B) *In vitro* SUMO assays using CBP proteins, as described in the legend to Fig. 2B. (C) Immunostaining of FLAG-CBP polypeptides in fixed COS-1 cells, using anti-FLAG antibodies. The images shown are typical nuclear distribution patterns for each construct. The scale bar is equivalent to 2 μm.

To determine if other sequences in CBP contribute to SUMOylation, *in vitro* SUMO assays were performed using a series of C-terminally truncated FLAG-CBP proteins that sequentially deletes functional domains such as the SID (1–1901), CH3 (1–1458), CH2/HAT/Bromo (1–1100) and CH1/KIX (1–507) domains (Fig. 3A). Surprisingly, only full-length CBP (Fig. 2A) and to a lesser extent CBP 1–1901 (Fig. 3B) were SUMOylated *in vitro*, despite the presence of the SUMO targeted region in CBP 1–1458 and 1–1100. This suggests that sequences in the C-terminus of CBP may be important to facilitate SUMOylation. Transient expression of these constructs in COS-1 (Fig. 3C) or HEK293 cells (not shown) revealed that while all of the truncated CBP proteins were nuclear, only full-length CBP showed a strong association with nuclear bodies. Thus, sequences at the C-terminus of CBP are required both for efficient Ubc9-dependent SUMOylation of CBP, and for targeting of CBP to nuclear bodies.

### SUMO-defective CBP shows reduced mobility in the nucleoplasm

**Fig. 4:**
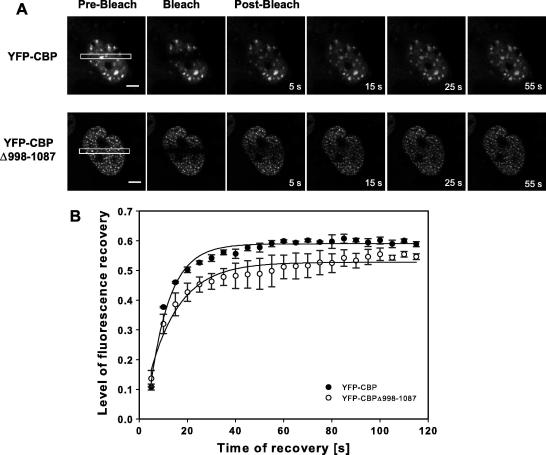
*SUMOylation influences the mobility of CBP in the nucleoplasm*. Fluorescence recovery after photo-bleaching (FRAP) analyses was performed on COS-1 cells expressing YFP–CBP or YFP–CBPΔ998–1087. (A) Representative images for YFP fusion proteins shown before bleaching (pre-Bleach), immediately after the bleaching (bleach) and at the indicated times thereafter (post-bleach). The bleached region of interest is boxed. Scale bar represents 5 μm. (B) Fluorescence recovery curves for YFP–CBP (black squares) and YFP–CBP (grey circles) showing the kinetics of redistribution post-bleaching. Best fit curves were generated using Sigmaplot software and standard error of the mean is shown for each time point.

To determine whether SUMO modification of CBP alters its dynamic mobility in the nucleoplasm, FRAP experiments were performed using COS-1 cells expressing YFP–CBP or YFP–CBPΔ998–1087. Cells showing similar levels of expression and typical subcellular distributions of the recombinant YFP proteins were selected for photo-bleaching. While YFP–CBP was found to accumulate in nuclear bodies as before (Fig. 3A, upper panels) YFP–CBPΔ998–1087 in live cells showed an accumulation in microspeckles (Fig. 3A, lower panels), similar to that observed in fixed cells (Fig. 2C). Photo-bleaching was performed on a defined region of interest as described in ‘Materials and Methods’, under conditions where bleaching did not affect neighbouring YFP positive cells (data not shown). FRAP was performed on three individual cells each for YFP–CBP and YFP–CBPΔ998–1087, selecting cells showing typical nuclear distributions. As shown for representative cells in Fig. 4A, both YFP–CBP and YFP–CBPΔ998–1087 were able to repopulate the bleached areas. The mean fluorescence recovery half-time for YFP–CBP in COS-1 cells was determined as 19 s. However, YFP–CBPΔ998–1087 displayed a retarded mobility half-time (Fig. 4B) displaying a mean fluorescence recovery half-time of 45 s. Both the rate and extent of recovery were reduced, as shown by the plateau of recovery at a lower percentage of the total initial fluorescence (Fig. 4B). These results indicate a reduced mobility of the SUMOylation-defective mutant CBP in the nucleoplasm, and suggest that SUMO modification regulates the mobility of CBP in the nucleoplasm, perhaps by modifying its association with nuclear proteins, chromatin or macromolecular structures such as nuclear bodies.

## Discussion

Using confocal microscopy and live cell imaging techniques, we have shown here that following an initial diffuse nuclear localisation, YFP–CBP proteins gradually accumulate in nuclear bodies. This targeting to nuclear bodies is dependent on the region of CBP that is subject to SUMO modification, in addition to sequences in the C-terminus. A proportion of the nuclear bodies containing YFP–CBP were observed to move relatively large distances within the nucleoplasm (up to 1 μm), consistent with a previous report that a proportion of GFP-SP100 nuclear speckles display mobility within the nucleus [15]. We noted that some of the YFP–CBP speckles form at the nuclear periphery and appear to move along the axis of the nuclear membrane. This phenomenon was observed in all three of the YFP–CBP expressing cells imaged in Fig. 1, and in other time-lapse experiments (data not shown). Failure to observe this in fixed cells is likely to be due to the collapse of the cellular integrity under conditions of fixation.

The juxtapositioning of CBP-containing nuclear bodies near the nuclear membrane is of interest given recent findings of functional interactions between CBP, nucleoporins and nuclear transport proteins [17,18]. Indeed nuclear transport proteins have been shown to bind chromatin and function in gene regulation [19,20]. CBP has been reported to interact directly with nucleoporins NUP98 and NUP153 via the FG repeats, which also mediate its interaction with NUP98-HOXA9 fusion protein associated with acute myeloid leukaemia [21]. We also reported the isolation of NUP93 heptad repeat domain in a yeast two-hybrid screen for proteins that bind the C-terminus of CBP [18]. Moreover, CBP forms a complex with importin-α and its exportin (CAS) via a LLXXLXXLL type motif in CAS, resulting in acetylation of importin-α [18], as necessary for its recycling to the cytoplasm [17]. Interestingly, another lysine acetyltransferase (MOF) has also been reported to associate with nuclear pore components, in complexes targeted to the nuclear periphery, which are involved in transcriptional regulation of dosage compensation in drosophila and mammalian cells [22].

Freely mobile proteins quickly recover almost 100% fluorescence in FRAP experiments, whereas less mobile proteins such as PML show very long recovery times, and plateau at a level of fluorescence less than 100% [2]. In our FRAP experiments, the time taken for YFP–CBP to recover 50% fluorescence in COS-1 cells was 19 s (Fig. 4B). However, the SUMO-defective CBP protein exhibited a longer recovery time, suggesting that the SUMOylated CBP proteins have increased mobility in the nucleoplasm. SUMOylation may decrease the residency time of CBP on chromatin at active gene promoters/enhancers. Alternatively, SUMO modification may alter the ability of CBP to interact with other nuclear proteins.

In summary, we have demonstrated here that the gradual accumulation of recombinant YFP–CBP proteins in nuclear bodies in live cells is dependent on a domain that contains the major SUMO modification sites in CBP. This region, in conjunction with C-terminal CBP sequences, acts to facilitate association of CBP with nuclear bodies. Moreover, a CBP mutant lacking this domain showed altered coactivator activity in AML1-dependent reporter assays, and reduced mobility in the nucleoplasm in FRAP experiments. Thus, post-translational modification of CBP by SUMO proteins is likely to influence the mobility of CBP in the nucleoplasm, its ability to associate with nuclear proteins and accumulate in nuclear bodies, and its function as a transcriptional coactivator.

## Conflict of interest statement

The authors have no conflicts of interest to disclose.
